# Tennis Serve Biomechanics, Joint Load Mechanics and Overuse Injuries: A Narrative Review

**DOI:** 10.1186/s40798-026-01015-y

**Published:** 2026-04-10

**Authors:** Caroline Martin, Pierre Touzard, Loic Fourel, Babette M Pluim

**Affiliations:** 1 Univ Rennes, Inria, CNRS, IRISA-UMR 6074, Rennes, 35000 France; 2 Medical Department, Royal Dutch Lawn Tennis Association, Amstelveen, The Netherlands; 3 Section Sports Medicine, Faculty of Health Sciences, University of Pretoria, Pretoria, South Africa

**Keywords:** Tennis serve, Joint loadings, Kinetics, Injury, Pathomechanics, Material, Racket sport

## Abstract

**Background:**

The tennis serve is a fundamental stroke in a player’s performance during matches. But it may be also eminently traumatic as it causes very high joint mechanical loading which can lead to injury regardless of the player’s experience, sex or age. This narrative review aims to synthesize current scientific knowledge on the key factors contributing to changes in joint mechanical loadings during the tennis serve and its potential association with overuse shoulder, elbow, wrist and lower back injuries.

**Main Body:**

The databases PubMed, Google Scholar, ScienceDirect and SPORTDiscus were used to search the related literatures. The publications that evaluated joint mechanical loadings through 3D biomechanical serve motion capture or video analysis in tennis players of all ages, sexes and competitive levels, with or without epidemiological monitoring of injuries were included. Most studies have involved small populations of expert able-bodied male players and focused only on the flat first serve. The results show that multiple factors – including skill level, energy flow, waiter’s serve technique, temporal kinematics, fatigue and racket specifications - seem to influence upper limb and lower back loadings, thereby influencing injury risks. The impact of serve type (kick, slice or flat), leg drive and backswing style (full or abbreviated) on joint loadings remains unclear.

**Conclusions:**

Teaching proper serving technique, choosing appropriate equipment and training conditions can limit the intensity of forces and moments applied on the joints and therefore decrease the risk of injury. However, further research is needed on this topic, especially in under-represented populations such as female, lower-level, juniors, and wheelchair tennis players.

## Key Points

- Various factors, including skill level, energy flow, pathomechanics, racket characteristics, and serving conditions influence lower back and upper limb joint loadings during the tennis serve, impacting the risk of overuse injuries while stance technique has no effect.
- The impact of serve type (kick, slice or flat) and backswing style (full or abbreviated) on joint loading, as well as whether an efficient leg drive reduces load, remains uncertain. These questions require further research and debate.
- Most studies have involved small populations of expert, able-bodied male players. To fill the gaps and improve the quality of evidence, future research should include broader player demographics, particularly female athletes, lower-level players, juniors, and wheelchair players.

## Background

The serve has undeniably grown in importance within tennis over the years, playing a decisive role in performance and match outcomes at the highest level of the sport [1, 2]. Professional male and female tennis players can generate impressive ball speeds (around 53 and 44 m.s^− 1^, respectively) and spin rates (ranging from 127 to 337 rad.s^− 1^) while maintaining precise placement during the serve, giving them a strategic advantage in the rally from the very first shot [3, 4]. In elite wheelchair tennis, serving is even more critical, as over 40% of points are decided within 1 or 2 strokes [5]. In professional matches, serves account for 21% to 25% of all shots hit by both players [6], with players performing between 90 and 160 serves per match [7]. These numbers vary based on factors such as age, sex, and match format (best-of-3 or best-of-5 sets).

The serve is also the most physically demanding and traumatic stroke in tennis [8]. Its physical toll is attributed to both the high repetition rate and the biomechanical complexity of the motion. The serve is a highly complex ballistic motion that requires precise intersegmental coordination and involves extreme muscular activity, joint ranges of motion and segmental velocities. Its traumatic nature is well-documented. A 2-year survey of 2633 tennis players found that 25% of them identified the serve as the most physically demanding and injury-prone stroke [9]. Epidemiological studies among NCAA tennis players between 2014 and 2019 further highlight the serve’s role in injury prevalence, accounting for a higher proportion of reported injuries (10.3–12.0%) compared to other strokes, such as the forehand (6.4–8.0%), backhand (3.0-5.2%), smash (0.3-2.0%), and drop shot (1.0-1.2%) [10, 11]. Similarly, wheelchair players also recognized the serve as the most painful stroke and a leading cause of their injuries [12].

The serve is specifically associated with overuse injuries affecting the lower back, shoulder, wrist and elbow [13–17]. Overuse injuries have traditionally been defined by the absence of a single, clearly identifiable traumatic event [18]. Research reports a wide range of injury prevalence among tennis players of different ages and skill levels. Shoulder injuries, for example, occur in 3% to 36% of able-bodied players [19, 20] and in 55% to 70% of elite wheelchair players [12, 21]. Common shoulder injuries include superior labral anterior-posterior (SLAP) lesions, rotator cuff injuries, biceps tendinopathy and shoulder instability [13, 22]. Lower back injuries, such as paraspinal muscle strain, ligament sprain, intervertebral disc degeneration, and pars interarticularis stress reactions or spondylolysis [13], range from 3 to 72% in able-bodied players [19, 23]. Tennis players are often prone to elbow injuries such as lateral epicondylitis (called “lateral tennis elbow”) and flexor-pronator tendinitis [13]. The overall incidence of lateral tennis elbow is estimated to be between 35% and 51% [15]. Additionally, some evidence suggests that the problem of wrist pain and injuries (e.g., De Quervain tenosynovitis, triangular fibrocartilage complex tear and intersection syndrome [24, 25]) in tennis players has increased between 1986 and 1995 and 2014–2015 [26].

Although direct comparisons between epidemiological studies are challenging due to methodological differences, these numbers highlight the importance of musculoskeletal injuries across all age categories and skill levels, from professionals and collegiate athletes to amateur adults and junior players [10, 11, 15, 27]. These injuries can disrupt training, competition, and long-term progression in the sport and are a major reason for tournament withdrawals. Furthermore, they can also affect mental health and, in the case of prolonged injury, may even end a high-level career [28].

To better understand the onset of sports injuries, several multifactorial injury aetiology models have been developed and refined in recent years [29–31]. These models constantly highlight that musculoskeletal sport injuries result from a complex interplay of intrinsic and extrinsic risk factors. These factors include age, sex, skill level, muscle weakness and imbalances, playing surface, training and competition loads, improper equipment, poor biomechanical movement patterns, psychosocial factors, and excessive mechanical loadings. While some risk factors, such as age and sex, are unmodifiable, others – such as biomechanical patterns and technical failings, equipment, training and competition characteristics can be adjusted to reduce injury risk.

Mechanical loading refers to the forces exerted on biological tissues or structures, originating from either external sources (e.g., collision with an opponent) or internal sources (e.g., muscle contractions pulling on bones or joints) [32]. This loading generates stress within the tissue, which can be classified as tensile or compressive when forces act perpendicular to the joint, shear when forces act parallel to the joint, or torsional when rotational moments are applied. Injury occurs when the stress induced by mechanical loading exceeds the tissue’s ultimate strength, leading to structural failure, or when repeated sub-maximal loadings surpass the tissue’s fatigue resistance over time [32]. Repetitive exposure to tensile, compressive, shear and torsional stresses can lead to microtrauma and overuse joint injuries [14]. Indeed, overuse injuries are generally considered to be repetitive microtrauma to the tissues [33]. In overhead sports motions, excessive internal mechanical load has very often been put forward as a major risk factor for shoulder, elbow, and lower back injuries. Consequently, traditional methods in overhead motion biomechanics have primarily focused on evaluating internal mechanical loading by estimating the peak values of forces and moments applied to the joints during the serve. This is typically achieved using 3D motion capture and the inverse dynamics approach [34]. The long-standing theory on the relationship between joint loadings and the incidence of overuse injuries in overhead sport motions was first confirmed in baseball [35]. Indeed, in a population of 25 pitchers whose injuries were prospectively recorded over three seasons, it has been reported that increased shoulder and elbow loadings were associated with increased elbow injury [35]. Moreover, Keeley et al. (2012) showed that peak shoulder proximal force was significantly correlated with shoulder pain in young baseball pitchers. Their logistic regression model showed that for every 1 N increase in peak proximal force, there was a corresponding 4.6% increase in the likelihood of shoulder pain [36]. In tennis, research has shown that during the backswing phase, joint moments and forces in the upper limb are relatively low, whereas the acceleration phase generates high shoulder internal rotation and horizontal adduction moments, along with significant elbow extension and varus moments [37]. During the follow-through, which serves to decelerate the racket and dominant arm, substantial shoulder compressive forces, external rotation and horizontal abduction moments, as well as high elbow and proximal forces, have been observed [38, 39]. A study involving 20 male elite players confirmed the relationship between excessive joint loading and serve-related injuries by combining motion capture analysis with a 2-season prospective registration of upper limb overuse injuries. Seven of the nine peak joint loadings analyzed during the serve were significantly higher in the injured group compared to the non-injured group [40].

This review aims to synthesize the current scientific knowledge on the key factors influencing joint mechanical loadings during the tennis serve and its potential association with overuse shoulder, elbow, wrist and lower back injuries. This review will address the following question: what factors contribute to increased mechanical joint loadings during the serve, thereby increasing the risk of injury? Additionally, the review seeks to identify gaps in the current understanding of joint loadings and their role in serve-related injuries, encouraging further biomechanical research in this area.

## Methodology

A retrospective, citation-based methodology was applied to search for English and French literature published since 1980 that examined upper limb joint loading during the tennis serve and its implications for injury. Peer-reviewed journal articles were the primary source. A structured search was conducted in PubMed, Google Scholar, ScienceDirect and SPORTDiscus with the following keywords in the title and abstract: tennis serve AND/OR joint loadings AND/OR joint kinetics AND/OR joint torques AND/OR joint moments AND/OR joint forces. Hand-searching of key journals and citation tracking of the retrieved articles was also conducted to identify additional relevant articles. Patient consent and ethics committee approval were not required. Articles had to meet the following criteria to be included in this review :

- Evaluation of serve biomechanics through 3D motion capture or video analysis in tennis players of all ages, sexes and competitive levels.
- Assessment of joint mechanical loadings during the tennis serve with or without implementation of epidemiological monitoring of injuries.
- Full text written in English or in French.
- Published in a peer-reviewed journal.

Exclusion criteria consisted were as follows:

- Case-reports, editorials, letters to the editor, commentaries, or literature reviews.
- Cadaveric studies.

Two authors independently assessed studies identified by the search criteria for applicability to inclusion/exclusion criteria. If the title and abstract did not provide sufficient information to determine whether eligibility criteria were met, the study was included for subsequent independent full-text review. Following the screening process, a total of 17 studies were included in this review. Additionally, data from two PhD theses that fulfilled the inclusion criteria were included [41, 42]. To structure the review with regard to the objective (identifying the factors contributing to increased mechanical joint loading during the serve and thereby increasing the risk of injury), the authors grouped the studies into main subtopics based on the factors influencing joint loadings highlighted in the studies.

## Results

### Identified Main Subtopics

The authors classified the different studies into the following seven subtopics based on the possible factors influencing joint loadings : sex (*n* = 1), skill level (*n* = 1), pathomechanical factors (*n* = 6), type of serve (*n* = 3), wheelchair players (*n* = 2), fatigue (*n* = 1), environment (*n* = 6). One study was positioned in 2 subtopics (sex and pathomechanical factors).

### Sex

A study conducted during the Sydney 2000 Olympics on 20 professional male and female players found that the higher ball speed with male players (50.8 vs. 41.4 m.s^-1^) was associated with greater shoulder internal rotation, horizontal adduction, elbow varus moments, and higher shoulder compressive forces [43]. However, shoulder anterior forces were similar across all players, regardless of sex. This study remains the only one to date comparing joint loadings between men and women during the serve. These findings highlight the lack of research on joint loadings in female players and underscore the need for further investigations to better understand sex-specific biomechanical characteristics and injury risk.

### Skill Level

Only one study has examined the effect of skill level on upper limb joint loadings during the serve. Based on motion captures of 11 male professional players (with an International Tennis Number 1) and seven male advanced players (with an International Tennis Number 3 or 4,) Martin et al. [39] showed that advanced players experienced significantly higher shoulder inferior and anterior forces, shoulder horizontal abduction moment, and elbow medial force, while professional players generated significantly higher ball speeds and elbow proximal forces. No significant differences in wrist loadings were observed between the two groups [39]. Since advanced players are subjected to higher joint loadings, the results suggest that they appeared more susceptible to high risk of shoulder and elbow injuries than professionals, especially during the cocking and deceleration phases of the serve.

Based on the results obtained, the authors formulated the following hypotheses. The increased anterior shoulder force observed in advanced players during the cocking phase may elevate their risk of “pathologic shoulder laxity” and glenoid labrum injuries compared to professionals. Additionally, their higher shoulder horizontal abduction moment during the deceleration phase could place them at greater risk of rotator cuff injuries, often associated with postero-superior impingement. Furthermore, the increased elbow medial force in advanced players during the cocking phase may increase their susceptibility to ulno-humeral injuries. For professional players, the greater proximal forces along the radial aspect of the elbow during the deceleration phase might lead to the development of osteochondral lesions or osteochondritis dissecans in the capitellum [44]. The higher joint loadings in advanced players compared to professionals could be attributed to less efficient serving techniques, potentially leading to increased injury risk. However, no injury data were collected in this study, which is a limitation.

### Pathomechanical factors

An efficient server is one who maximises ball speed while minimising joint loadings [39]. Any kinematic or temporal pattern that significantly increases joint loading without increasing ball speed is considered a ‘‘pathomechanical’’ technical failing. Several pathomechanical factors have been identified for the tennis serve.

### Stance Technique and Leg Drive

In junior male and female expert players, similar levels of shoulder, elbow, and wrist moments and forces were observed between the foot-up (back foot is moved forward next to front foot for push-off) and foot-back techniques (feet remain separated and fixed until push-off) [41] indicating an identical injury risk for these two techniques. This insight is valuable for tennis coaches, physiotherapists and medical doctors who can confidently recommend either technique without increasing overuse injury risk. Reid et al. (2008) further compared three types of lower-limb involvement patterns among twelve high performance male players [45]: the foot-up serve, the foot-back serve, and minimal leg drive serve (characterized by reduced ranges of front and rear knees extension and a limited peak of back knee extension velocity). While ball speed was significantly reduced for the minimal leg drive serve, peak shoulder anterior force and peak internal rotation moment were comparable across the three serves. Consequently, the minimal leg drive does not appear to be more traumatic for the shoulder but reduces serve efficiency (lower ball speed but identical shoulder loadings).

Elliott et al. (2003) found that players with a limited front knee flexion (< 10°) at maximal shoulder external rotation generated similar ball speeds but experienced higher shoulder internal rotation, elbow varus and flexion moments compared to players with greater front knee flexion (> 10°) [43]. These findings suggest that ineffective leg action during the serve may be a pathomechanical factor increasing the risk of shoulder and elbow overuse injuries. However, assessing leg action efficiency solely based on front knee flexion angle at maximal shoulder external rotation constitutes a limitation, as it provides an incomplete understanding of the overall contribution of the lower limbs to the injury risks.

While the studies by Touzard (2021) [41], Elliott et al. (2003) [43] and Reid et al. (2008) [45] reported interesting findings concerning stance technique and leg drive, they have not implemented prospective injury follow-up. Moreover, these studies have primarily focused on basic leg drive variables, such as foot stance, knee flexion/extension angles and velocities. Recently, Fourel et al. (2024) demonstrated that more complex force-time curve variables (mean vertical ground reaction force, net vertical impulse during the concentric phase and the maximal value of the vertical power) allow a full understanding of the serve leg drive [46]. In baseball, Aguinaldo and Nicholson (2022) confirmed the relationship between force-time curve variables and the peak elbow valgus moment in a sample of high school and collegiate pitchers [47]. They reported that front leg medial impulse, back leg propulsive ground reaction force and back leg medial impulse were significantly and negatively correlated with the peak elbow valgus moment. In tennis, further research combining the measurement of these complex variables, upper limb joint loadings and prospective injury recording is needed to provide a deeper understanding of the role of leg drive in injury prevention.

### Racket Motions During the Backswing and the Cocking Phases

**Fig. 1:**
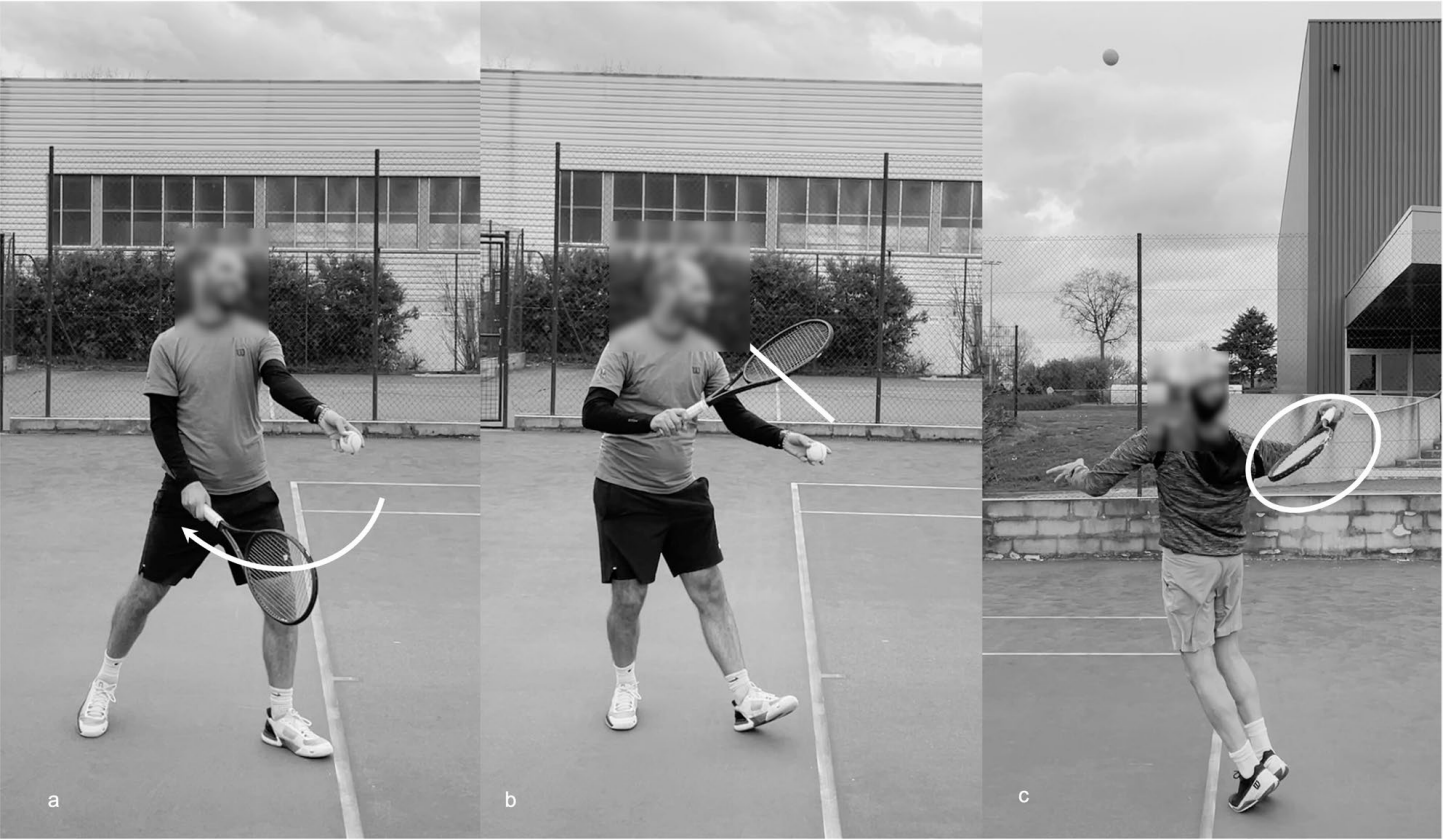
Full (**a**) and abbreviated (**b**) backswings; waiter’s serve posture (**c**)

Elliott et al. (2003) compared upper limb joint loadings between the full and abbreviated serve backswings [43]. The abbreviated backswing is characterized by a shorter, more direct, sideways racket ascent to the trophy position (that corresponds to the racket high point during the preparation phase of the serve), while the full backswing involves a downward and backward trajectory of the racket before moving towards the trophy position (Figs. 1a − 1b). The study found that players with an abbreviated serve backswing generated similar ball speed, shoulder internal rotation, elbow varus and flexion moments compared to those with a full swing. However, there was a trend toward higher anterior shoulder force in players with an abbreviated backswing (*p* = 0.05). This could potentially increase the risk of excessive humeral head translation, shoulder instability and impingement syndromes [48, 49]. According to Elliott et al. (2003), the full backswing may be a preferred service technique from a loading perspective. However, this conclusion is questionable given that only one out of six shoulder and elbow loadings showed a significant difference.

The waiter’s serve corresponds to a specific racket posture, in which there is a very open racket face (racket face parallel to the ground) during the cocking phase (Fig. 1c), frequently observed in young players and beginners, and is often considered a technical error by tennis coaches. It has been identified as a pathomechanical factor in a study involving 18 male junior elite players all belonging to the same national training center [50]. No significant difference in ball speed was observed between players exhibiting the waiter’s serve and those who did not. Players with the waiter’s serve posture exhibited significantly higher shoulder internal rotation and elbow varus moments, as well as increased wrist proximal and anterior forces. As a result, their theoretical risk of shoulder, elbow and wrist injuries was higher. However, these biomechanical findings were not fully supported by the epidemiological results obtained during a 12-month period following the motion capture. Indeed, no significant difference was found between the two groups regarding the incidence or severity of upper limb injuries. Only a trend was observed, suggesting that players using the waiter’s serve might be more prone to elbow injuries.

### Temporal Factors

Martin et al. (2013) identified additional pathomechanical factors relative to timing motions in 20 male expert tennis players [40]. Their serves were recorded with an optoelectronic motion capture system and the players were then followed during two seasons to identify overuse injuries of the upper limb. During this period, 12 overuse upper limb injuries including shoulder labral tears, rotator cuff tendinopathies, type 2 SLAP lesions, elbow and wrist tendinopathies were reported by 11 players. Correlation analyses showed that the earlier the initiation of shoulder external rotation relative to shoulder horizontal adduction, the higher the shoulder anterior force and horizontal abduction moment, and the lower the ball speed. Injured players, who maintained their arm in horizontal abduction for too long (4.8% of the serve duration) during the shoulder external rotation phase, experienced increased shoulder anterior (+ 13%) and inferior (+ 29%) forces, elbow medial force (+ 15%), shoulder horizontal abduction (+ 27%) and elbow flexion moments (+ 14%) and reached lower ball speed (-11.5 km.h) in comparison with non-injured players. This phenomenon, known as “hyperangulation”, is recognized for its detrimental effects on the shoulder anterior capsule [51], contributing to anterior shoulder instability and anterior labral lesions. It is also a key factor in internal impingement [52] which results from a translation of the humeral head relative to the glenoid [53], and can lead to rotator cuff tears, shoulder tendinopathies, and labral lesions.

Additional findings from Martin et al. [54] revealed that the later the peak angular velocities of the trunk transverse and sagittal rotations, upper torso, and pelvis longitudinal rotations, the higher the upper limb joint loadings and the lower the ball speed. Non-injured players who reached maximal trunk rotation velocities earlier were able to maximize ball speed while minimizing upper limb joint loadings. This suggests that an optimal energy flow from the trunk to the shoulder at the right moment within the kinetic chain is key to both performance and injury prevention. Conversely, improper or delayed peak angular velocity timings in the trunk, the pelvis, and upper torso may lead to energy loss, preventing efficient energy flow to the dominant upper limb. This loss of energy would force players to compensate by increasing joint loadings, and consequently expose them to a greater risk of upper limb injuries [54].

### Energy Flow

The relationships between energy flow during the tennis serve, and overuse injuries have been assessed in a study involving 19 high level players [54]. The serves were recorded with an optoelectronic motion capture system. The forces and torques of the upper limb joints were calculated from the motion captures by use of inverse dynamics. The amount of mechanical energy generated, absorbed, and transferred was determined by use of a joint power analysis. Energy flow quality was defined as the ratio between the mean rate of energy leaving the trunk during the cocking phase and the mean rate of energy reaching the hand and racket during the acceleration [54]. Then, the players were prospectively followed during 2 seasons to identify upper limb overuse injuries with a questionnaire. During this period, 6 players reported shoulder tendinopathy such as rotator cuff tendinopathies, labral tears, or type 2 SLAP lesions. Moreover, 5 players suffered from elbow tendinopathy (medial or lateral tennis elbow), and 1 had a wrist tendinopathy. The indicator of energy flow quality was significantly higher for non-injured players (88.1 ± 16.9%) compared to those who had sustained injuries in the dominant upper limb joints (71.1 ± 15.0%) [54]. Moreover, the indicator of energy flow quality was moderately correlated with ball speed, and weakly to moderately correlated with shoulder proximal, anterior, and inferior forces, elbow anterior and medial forces, and shoulder horizontal adduction and elbow flexion moments. In other words, the players with high quality of energy flow from the trunk to the racket are those with the highest ball velocities and the lowest upper limb joint loadings. While this study has the merit of confirming that energy flow influences upper limb loadings and injury risks, it did not take into account the entire kinematic chain from the legs to the racket.

### Type of Serve (Slice, Kick and Flat)

In tennis, there is a widespread belief that the kick serve is more traumatic than the slice and flat serves [55], leading coaches to delay learning the kick serve after the age of 13 in growing players [56], or not to recommend the kick serve in players with injury problems. This hypothesis was reinforced by Abrams et al. (2013) who reported that the total force and moment experienced in the lower back by seven advanced male tennis players were significantly higher for the kick serve than for the flat serve [57]. Additionally, a trend towards significance was observed for total lower back moment with higher values for the kick serve compared to the slice [57]. While these results suggest that the kick serve may be more conducive to back injuries, unfortunately, the authors calculated total lower back force and moment without differentiating them according to axes of rotation or directions of application. It is therefore quite impossible to link these loadings to the specific back injuries encountered by tennis players. Another comparison involving adolescent male players reported that, conversely, the flat serve induced significantly greater back flexion moment than the kick serve during the forward-swing phase, probably to hit the ball more inside the court [58]. Abrams et al. (2013) observed no significant differences in the elbow and wrist loadings between the three types of serves [57]. Peak of posterior shoulder force was significantly greater for the kick serve than for the others [57], which may increase the risk of posterior glenoid labrum microtrauma [36, 59]. Conversely, Reid et al. (2007) found no differences in shoulder loadings between the kick and the flat serves among twelve high-performance male players and two top-30 professionally ranked wheelchair players, except for an upward trend in mean compressive forces and peak external rotation moment after impact in the flat serve [38]. These discrepancies may be attributed to differences in motion capture methodologies, with a study using markerless tracking [57] and others employing marker-based tracking [38, 58]. Consequently, whether the kick serve imposes greater shoulder and lower back loadings than the flat serve remains an open question, warranting further research. This issue is particularly relevant given that the lumbar region is the site of most spinal injuries, especially lumbar pars interarticularis stress reactions [60], and radiological abnormalities in adolescent tennis players [61]. Furthermore, the appropriate age for introducing the kick serve in young players remains a topic of debate [62].

### Effect of Impairment in Wheelchair Tennis Players

To date, no study combined a biomechanical serve analysis and an epidemiological monitoring of injuries in wheelchair tennis players to identify the relationships between joint loadings and injury risks. However, Reid et al. (2007) compared joint loadings during the serve of two top-30 professionally ranked wheelchair players with different levels of impairment [38]. One player had an incomplete injury at the T12 level but a complete break at the L1 level, resulting in no lower limb function, while the other had an incomplete T10 spinal cord injury, allowing for more trunk and lower limb function, including the ability to walk unassisted for short distances [63]. The study found that shoulder loadings – including peak external and internal moments, mean compressive and distractive forces – were higher in the player with the less severe impairment. These findings suggest that the level of functional impairment may influence shoulder loadings during the serve.

In wheelchair tennis, players compete in either the open category (lower limb impairment only) or the quad category (combined upper and lower limb impairment). Comparisons between open and quad categories showed that peaks of shoulder horizontal flexion and internal rotation moments, wrist flexion and radial deviation moments were significantly higher in the open class [42]. While these findings provide valuable insights, the limited sample size (≤ 3 per group) constrains the scope and reliability of the conclusion. Future research should analyse larger cohorts of wheelchair players to enhance the quality of evidence and better understand the unique biomechanical demands and injury risks in wheelchair tennis.

### Fatigue

Understanding how muscular fatigue influences serve biomechanics, joint loadings and performance is of significant interest for tennis players, coaches, and medical practitioners. To explore this, a study recorded the serves of eight advanced male tennis players using a motion capture system before, midway through, and after a 3-hour match [64]. The results revealed a significant reduction in ball speed and all shoulder loadings from the start to the end of the match, whereas only four out of six elbow loadings and three out of five wrist loadings showed similar decreases. For Martin et al. (2016), it is unclear whether the significant changes in joint loadings were a direct result of fatigue that occurs with extended play or if the body adopted protective mechanisms to minimize the risk of injury over the course of a match or the result of these two phenomena [64]. In this study, shoulder loadings are most affected by fatigue, maybe because this is the joint that contributes most to racket speed at impact [65]. These results suggest that compensatory mechanisms at various joint levels may act to delay the effects of fatigue and try to maintain an efficient level of play; however, more work is needed to confirm and understand these preliminary findings. Moreover, in official junior tennis competitions, a significant increase in the medical withdrawal rate has been reported beyond the fourth match of the tournament [66]. With the advancement of markerless motion capture systems, future studies will be able to reproduce this type of research in real competition settings to identify the evolution of joint loadings not only during a single match but throughout a tournament. This would allow for a more comprehensive analysis of how fatigue influences joint loadings and injury risk during successive matches across different skill levels, age groups and sexes.

### The Environment of the Adult Tennis Player

#### Influence of Interrelated Racket Specifications

Regarding the tennis player’s environment, the racket is often cited as a potential risk factor for injury [67]. A tennis racket is characterized by several characteristics: length, mass, head size, beam width (thickness of the racket frame), stiffness, position of the balance point and moments of inertia (MOI) (swingweight MOI about the medio-lateral axis, polar MOI about the longitudinal axis and the lateral or spinweight MOI about the normal or antero-posterior axis). A number of these have been investigated in the literature (Table 1).

**Table 1:** Summary of studies examining the impact of racket characteristics on serve joint loadings

Reference	Number of players	Sex	Age (years)	Level	Features of the tested rackets	Main results
Rogowski et al. [72]	8	Male	Adults 26.7 ± 4.9	ITN 3 Advanced	Two different expert rackets in terms of polar MOI (0.00152 vs. 0.00197 kg.m^− 2^) but identical string tension (250 N), mass (0.327 kg), balance (0.336 m) and swingweight MOI (0.0339 kg.m^− 2^)	Increase in polar MOI leads to: • no significant difference in ball speed and racket resultant velocities before impact • increases in peak shoulder extension and abduction moments, elbow extension, valgus and supination moments in the cocking phase • increases in peak wrist extension and radial deviation moments in the forward swing • decreases in peak shoulder adduction, elbow pronation and wrist external rotation moments in the follow-through
Wang and Huang [71]	4	Female	Adults 22.5 ± 2.7	Advanced	Four different rackets in terms of swingweight MOI and balance (0.0285, 0.0309, 0.0336, 0.0415 kg.m^− 2^ and 0.298, 0.334, 0.350 and 0.376 m) but same mass (0.320 kg)	Highest swingweight MOI and highest balance lead to: • decrease in racket head speed at impact • increase in net wrist force (in comparison with lowest swingweight MOI)
Miao et Zhou [69]	8	Male	Adults 25.3 ± 3.4	Intermediate 5.6 ± 1.2 of competitive tennis experience	Three different rackets in terms of balance, mass and swingweight MOI Head light vs. even-balanced vs. head-heavy	The head heavy racket leads to: • decreases in shoulder internal rotation and elbow extension velocities • increases in shoulder moments (adduction, abduction, flexion, extension, internal rotation, external rotation) • increases in wrist, elbow and shoulder forces
Creveaux et al. [68]	5	Male	Adults 25.3 ± 3.4	ITN 3 Advanced	Three rackets for experts mainly different in terms of mass and MOI	Decrease in racket mass and increase in balance and polar MOI lead to: - no significant difference in ball speed - increases in shoulder internal and external rotation moments, respectively during the acceleration and the follow-through phases
Touzard et al. [73]	9	Five male and four female	Children 9.9 ± 1.0	Intermediate to recreational (ITN 6–9)	Three beginner rackets different in terms of length (23, 25 and 27 inches), mass, balance, head size and MOI	Increase in racket length leads to: • decreases in ball speed and maximal racket head velocity • no significant difference in % of serves in • increases in shoulder internal rotation and abduction moments, and elbow varus moment

MOI: moment of inertia, ITN: international tennis number

Creveaux et al. (2013) analyzed the influence of three different rackets on peak shoulder moments during the flat serve in five advanced tennis players [68]. The rackets varied in mass, balance and MOI, yet all fell within the category for expert players (Table 2).

**Table 2:** Characteristics of the strung rackets tested in Creveaux et al. (2013) [68]

Mass (kg)	Racket A	Racket B	Racket C
	0.340	0.360	0.387
Balance (m)	0.331	0.321	0.326
Swingweight (kg.m^− 2^)	0.0346	0.0331	0.0368
Lateral MOI (kg.m^− 2^)	0.0364	0.0346	0.0385
Polar MOI (kg.m^− 2^)	0.0017	0.0014	0.0016

MOI: moment of inertia

Ball speed and serve phase durations (downward acceleration, upward acceleration and follow-through) remained unchanged regardless of the racket. However, racket A generated the highest peak of shoulder internal and external rotation moments during the acceleration and the follow-through phases, respectively. This suggests that serving with the lightest racket with the highest balance and polar MOI increases biomechanical loadings on the shoulder without improving ball speed, compared to heavier rackets with lower balance and polar MOI. However, due to the interrelated specifications of the tested rackets, it remains unclear whether the observed effects are due to mass, balance, or MOI.

#### Influence of Mass and Balance

A recent study evaluated the influence of three racket types (head-light, even-balanced and head- heavy) on upper limb joint moments during the flat serve among 8 competitive tennis players using a machine learning-based approach (Table 3) [69].

**Table 3:** Characteristics of the rackets tested in Miao and Zhou (2024) [69]

Mass (kg)	Racket A Head light	Racket B Even-balanced	Racket C Head-heavy
	0.300	0.320	0.340
Balance (m)	0.330	0.340	0.355
Swingweight MOI (kg.m^− 2^)	0.0280	0.0290	0.0310
Polar MOI (kg.m^− 2^)	0.0010	0.0011	0.0013

MOI: moment of inertia

Results indicated that the head-heavy racket generated the highest shoulder moments (adduction, abduction, flexion, extension, internal rotation, external rotation), wrist, elbow and shoulder forces, whereas the head-light racket produced the lowest. The even-balanced racket showed intermediate shoulder moments and upper limb joint forces, with values between the two other rackets. Although ball speed was not assessed, the highest shoulder internal rotation and elbow extension velocities, key contributors to the racket head velocity at impact [70], were observed with the head-light racket, while the lowest were recorded with the head-heavy racket. Thus, the authors concluded that the head-light racket minimized upper limb joint loadings while enhancing performance.

#### Influence of the Swingweight MOI

A study performed on four expert female players examined peak upper limb joint loadings using 4 rackets with different swingweight moments of inertia (MOI) and balance points (0.0285, 0.0309, 0.0336, 0.0415 kg.m^− 2^ and 0.298, 0.334, 0.350 and 0.376 m, respectively) [71]. Although the rackets had identical mass (0.320 kg), the mass distribution was modified by adding an extra 0.04 kg at different positions of each racket. Findings showed that a higher swingweight MOI led to a decrease in racket head velocity at impact and a significant increase in wrist joint reaction force. Consequently, rackets with high swingweight MOI and balance points (> 0.0336 kg·m², > 0.350 m) should be avoided to reduce the risk of wrist injuries in expert female players. However, due to the study’s small sample size (four players), further research is necessary to confirm these findings and strengthen the evidence base for racket design recommendations.

#### Influence of the Polar MOI

Rogowski et al. evaluated the effects of racket polar MOI on peak upper limb joint loadings in eight competitive male adult tennis players [72]. The study compared two rackets that were identical in string tension (250 N), mass (0.327 kg), center of mass (0.336 m), and swingweight (0.0339 kg·m²), but with different polar MOI (0.00152 vs. 0.00197 kg·m²). While ball speed, racket velocity and serve phase durations were unaffected, joint loadings varied significantly.

During the cocking phase, the racket with the highest polar MOI resulted in significantly greater peak shoulder extension and abduction moments, as well as increased elbow extension and supination moments. Rogowski et al. suggested that repeated exposure to elevated shoulder abduction moments during a match could contribute to supraspinatus muscle fatigue, compromising the maintenance of congruence between the humeral head and the glenoid cavity [72]. Additionally, the increased polar MOI led to a significant rise in elbow valgus moment during the cocking phase, which may overload bony and ligamentous structures. Indeed, the increased polar MOI of the racket could exacerbate the abutment of the posteromedial olecranon on the medial wall of the olecranon fossa and aggravate the valgus extension overload mechanisms, known to cause medial elbow injuries. It can also cause damage to the ulnar collateral ligament.

During the forward swing, peak shoulder and elbow moments remained unaffected by the racket’s polar MOI [72]. However, peak wrist extension and radial deviation moments increased significantly with the higher polar MOI, potentially elevating the risk of lateral epicondylitis pain. Conversely, in the follow-through, peak shoulder adduction, elbow pronation and wrist external rotation moments significantly decreased with higher polar MOI, suggesting reduced post-impact upper limb joint loadings and injury risks. However, since peak joint moments were lower in this phase compared to earlier phases, the benefits of a higher polar MOI remain debatable according to Rogowski et al. [72]. Overall, while higher polar MOI rackets may improve off-center shot stability, they appear to increase upper limb joint stress and injury risk in competitive male adult players.

### The Environment of the Young Tennis Player

#### Influence of the Racket Length

A single study examined the impact of three different racket lengths (23, 25 and ‘full-size adult’ 27 inches) and their characteristics on upper limb joint loadings during the serve in young intermediate and recreational tennis players [73] (Table 4).

**Table 4:** Characteristics of the rackets tested in Touzard et al. (2023) [73]

Length (m)	23 inches	25 inches	27 inches
	0.584	0.635	0.685
Unstrung mass (kg)	0.190	0.239	0.249
Unstrung balance (m)	0.275	0.305	0.340
Head size (cm^2^)	613	645	658
Swingweight MOI (kg.m^2^)	0.0159	0.0219	0.0308
Polar MOI (kg.m^2^)	0.0007	0.0009	0.0010

MOI: moment of inertia

Serve performance indicators (ball speed, maximal racket head velocity and percentage of serve in) were similar between the three rackets. However, significantly higher peaks in shoulder horizontal abduction, internal rotation moments, and elbow varus moment were obtained with the adult racket. The increased shoulder horizontal abduction and internal rotation moments elevate the risk of rotator cuff overuse injuries and shoulder tendinopathies, both common in young tennis players [74]. Additionally the higher peak of elbow varus moment may heighten the risk of tennis elbow, medial epicondylitis and medial epicondylar growth plate lesions, often encountered by young tennis players [74, 75]. Consequently, tennis coaches, parents, and medical staff are advised to delay transitioning young players towards adult rackets in order to protect their shoulder and elbow from potential injuries.

#### Influence of the Serving Conditions (Serve Distance and Net Height) on Young Players

A study of ten young intermediate competitive players (five boys and five girls) examined the effects of different serving distances and net heights using “green” balls [76]. Players served from 3 court setups corresponding to the “red”, “orange” and “green” courts, with distances of 6.40 m, 9.00 m and 11.89 m from the net, respectively [77]. The net height was 0.80 m for the red and orange courts and 0.91 m for the green court.

Results showed that peak shoulder and elbow forces and moments, and consequently the risk of overuse injury, were similar among the three serving conditions. However, ball speed was significantly lower when serving from the green court compared to the red court, with a similar trend observed between the green and orange courts. Young tennis players achieved greater ball speed without increasing joint loadings when serving from a shorter distance and a lower net height, suggesting greater efficiency under these conditions [76].

These findings highlight the potential benefits of modifying key environmental constraints – such as serve distance and net height – to enhance serve efficiency in young players. Tennis coaches, clubs and federations are encouraged to adapt these parameters to optimize skill development while minimizing injury risk. However, the impact of serving conditions on adults, especially beginners, remains an open question that warrants further investigation. Moreover, studies evaluating the effects of racket specifications and serve conditions on joint loadings in young tennis players did not conduct epidemiological monitoring of the participants, which constitutes a limitation that future studies will have to address [73, 76].

### Practical Implications

Our review highlights the importance of optimizing serve mechanics to minimize joint loadings and injury risks (Table 5). Coaches should adapt serving conditions (net height, serve distance, racket size) and emphasize efficient kinetic chain sequencing, timing of trunk rotations, energy transfer, and avoidance of pathomechanical factors such as shoulder hyperangulation or “waiter’s serve”. Strength and conditioning programs should aim to enhance the capacity of the entire kinetic chain to generate, transfer, and absorb mechanical energy efficiently. Medical staff should consider serve biomechanics and equipment characteristics when evaluating injury risk and designing rehabilitation programs.

**Table 5:** Summary of studies examining the relationships between key factors, joint loadings and injury risk for the tennis serve

Joint loading	Associated injury risks	Influencing factor	Factor with no influence	Still debated factor (small sample size, contradictory results)
Shoulder anterior force	Pathologic shoulder laxity and instability, anterior glenoid labrum injuries, impingement syndromes [48, 49]	Skill level [39] Type of backswing [43] Shoulder hyperangulation [40] Timing of trunk transverse, sagittal, pelvis longitudinal angular velocities [40] Energy flow quality [54] Fatigue [64]	Stance technique [45]	
Shoulder posterior force	Posterior glenoid labrum injuries [36, 59]			Type of serve (kick, slice, flat) [38, 57]
Shoulder compressive force	Posterior impingement syndromes	Sex [43] Fatigue [64]	Stance technique [45]	Type of serve (kick, slice, flat) [57] Level of disability (spinal cord injury) [63]
Shoulder internal rotation moment	SLAP lesion, posterior and subacromial impingements [79]	Sex [43] Waiter’s serve [50] Timing of trunk transverse rotation [40] Fatigue [64] Racket’s length [73] Racket’s mass and balance [72]	Stance technique [45]	Wheelchair category (open or quad) [42] Minimal leg drive [43, 45] Level of disability (spinal cord injury) [63]
Shoulder horizontal adduction moment	Rotator cuff injuries, shoulder postero-superior and subacromial impingements, SLAP lesion [79]	Sex [43] Energy flow quality [54] Fatigue [64] Racket’s mass and balance [72]	Stance technique [45]	
Shoulder horizontal abduction moment	Shoulder postero-superior impingement, rotator cuff injuries [80]	Skill level [39] Shoulder hyperangulation [40] Timing of trunk transverse, sagittal and upper torso angular velocities [40] Racket’s length [73] Racket’s mass and balance [72]	Stance technique [45]	
Elbow proximal force	Osteochondral lesions or osteochondritis dissecans in the capitellum [44, 80, 81]	Skill level [39] Fatigue [64]	Stance technique [45]	
Elbow medial force	Ulna-humeral injuries Chondral loss, posteromedial osteophytes, and oleocranon stress fractures [44, 81]	Skill level [39] Timing of trunk transverse, upper torso and pelvis longitudinal angular velocities [40] Energy flow quality [54] Fatigue [64]	Stance technique [45]	
Elbow varus/valgus moment	UCL sprain, medial epicondylitis, ulnar neuritis, stress fracture, capitellar osteochondral lesions [44, 80–82]	Sex [43] Waiter’s serve [50] Timing of trunk transverse rotation [40] Fatigue [64] Racket’s polar MOI [72] Racket’s length [73]	Stance technique [45]	Minimal leg drive [43, 45]
Back forces and moments	Spondylolysis, lumbar disc degeneration and herniation, lower back pain [83, 84]			Type of serve (kick, slice, flat) [38, 57]
Wrist flexion moment	Intersection syndrome, traumatic and tendinous wrist injuries and ulnar carpal impingement [25, 85, 86]	Fatigue [64] Racket polar MOI [72]		Wheelchair category (open or quad) [42]
Wrist radial deviation moment	De Quervain’s tenosynovitis, traumatic and tendinous wrist injuries [25, 85]	Fatigue [64] Racket polar MOI [72]		Wheelchair category (open or quad) [42]
Wrist forces	De Quervain’s tenosynovitis, triangular fibrocartilage complex lesions [25, 85, 86]	Waiter’s serve [50] Fatigue [64] Racket’s mass and balance [72]		Racket’s swingweight MOI [72]

MOI: moment of inertia, UCL: Ulnar Collateral Ligament, SLAP: Superior Labrum Anterior and Posterior

### Limitations

This review has several limitations that must be mentioned. First, the present work is a narrative review and not a systematic review or meta-analysis. Although a structured search strategy was applied, no formal risk-of-bias assessment or quantitative synthesis of the included studies was conducted. As a result, the strength of evidence supporting some conclusions may vary across subtopics. Second, a major recurring issue in several studies is the small sample size used, which reduces the statistical power and can limit the generalisability of the conclusions. Moreover, the methods for calculating joint loadings, processing and presenting them in a specific coordinate system (global system, joint coordinate system, proximal or distal segment coordinate systems) with directional signs varied widely across studies, leading to considerable discrepancies in the literature on the tennis serve. In some studies, some of these methodological elements were not even reported. Additionally, joint loadings were expressed in either absolute or normalised values, with normalisation methods varying based on body mass, height, their product, or ball speed, further complicating cross-study comparisons. Some studies focused on peak joint loading, while others analysed joint loading at key moments or as mean values during specific phases of the serve. These methodological choices in expressing intersegmental forces and moments can significantly influence the interpretation of data [78]. For greater consistency, future studies on joint loading during the tennis serve should follow the International Society of Biomechanics recommendations for reporting intersegmental forces and moments in human motion analysis [78]. Finally, while several studies have linked increased joint loadings to overuse injuries, only a minority combined biomechanical analyses with prospective epidemiological follow-up. In many cases, associations between mechanical load and injury risk remain theoretical and based on extrapolation from other overhead sports such as baseball.

### Additional Factors Requiring Further Investigation

Several factors potentially influencing joint loadings during the tennis serve remain unexplored. Grip characteristics, including grip type and grip strength, may affect force transmission and upper limb loading. Additional factors such as ball toss location, ground reaction force patterns, and trunk rotation mechanics may also have an effect on joint loadings and injury risk. Future studies integrating these variables with joint kinetics and prospective injury monitoring are needed to better understand injury mechanisms.

## Conclusions

This review synthesized current scientific knowledge on joint mechanical loadings during the tennis serve and their association with overuse injuries of the shoulder, elbow, wrist and lower back (Table 5).

Several key findings for coaches, physiotherapists, medical professionals, equipment manufacturers emerge as well as the following future research directions for scientists:

1. Joint loading and injury risk: Multiple factors – including skill level, energy flow, kinetic chain efficiency, waiter’s serve technique, temporal kinematics, fatigue and racket specifications - seem to influence upper limb and lower back loadings, thereby influencing injury risk (Table 5). Teaching proper serving technique, choosing appropriate equipment and training conditions that minimize loading could mitigate these risks. However, further research is needed to identify new pathomechanical factors that could help tennis coaches and sports medicine professionals identify players with increased risk of injury.
2. Limited research diversity: Most studies have involved small populations of expert, able-bodied male players. To fill the gaps and improve the quality of evidence, future research should include broader player demographics, particularly female athletes, lower-level players, juniors, and wheelchair players. Moreover, researchers should consider combining motion capture and prospective epidemiological monitoring.
3. Serve variations and joint loadings: Research has primarily analyzed joint loadings of the upper limb in the flat serve. The analysis of the kick and slice serves must be deepened to determine which imposes greater stress on the lower back and the upper limb joints.
4. In-game biomechanics: Most studies have been conducted in controlled laboratory settings using marker-based motion capture. Advancements in markerless motion capture technology could provide more accurate assessments of joint loadings in real match situations, providing a more comprehensive understanding of in-game biomechanics.
5. Racket characteristics: Concerning racket characteristics, increased moment of inertia (MOI) and balance position heighten upper limb joint loadings during the serve among intermediate and advanced adult players. However, clear gaps remain in the literature, particularly concerning professional players, beginners, and the effect of racket length, head size, beam width and stiffness. Notably, research has primarily focused on relatively heavy rackets (> 300 g strung), commonly used by advanced players, while data on lighter rackets (260–290 g strung) more suitable for beginners, are lacking. Future research should address these gaps to optimize racket recommendations for players of all levels.

Expanding research in these areas will provide more comprehensive guidelines for injury prevention, performance optimisation, and equipment selection, ultimately benefiting players, coaches, and sports medicine professionals.

## Data Availability

The datasets used and/or analysed during the current study are available from the corresponding author on reasonable request.

## Abbreviations

ITN: International tennis number
MOI: Moment of inertia
